# Eliglustat substrate reduction therapy in children with Gaucher disease type 1

**DOI:** 10.3389/fped.2025.1543136

**Published:** 2025-02-27

**Authors:** Noor Ul Ain, Armaan Saith, Audrey Ruan, Ruhua Yang, Aaron Burton, Pramod K. Mistry

**Affiliations:** ^1^ Department of Internal Medicine, Yale School of Medicine, New Haven, CT, United States; ^2^ Specialty Pharmacy, Yale New Haven Hospital, New Haven, CT, United States; ^3^ Department of Pediatrics, Yale School of Medicine, New Haven, CT, United States

**Keywords:** Gaucher disease (GD), precision medicine, substrate reduction therapy (SRT), eliglustat, lysosomal storage disease (LSD)

## Abstract

**Importance:**

Gaucher disease (GD) is a rare lysosomal storage disorder with limited treatment options for pediatric patients. Oral substrate reduction therapy (SRT) with eliglustat offers a potential alternative, particularly for those with barriers to enzyme replacement therapy (ERT).

**Objective:**

Evaluate the safety and efficacy of eliglustat SRT in pediatric patients with type 1 Gaucher disease (GD1), both as initial therapy and as a switch from intravenous ERT.

**Design:**

A prospective case series was conducted from 2017 to 2024.

**Setting:**

Yale's National Gaucher Disease Treatment Center, New Haven, CT, United States.

**Participants:**

Fourteen pediatric GD1 patients with significant barriers to receiving ERT.

**Intervention:**

Eliglustat SRT was dosed pharmacogenomically based on CYP2D6 metabolizer status.

**Primary outcomes and measures:**

Primary outcomes included safety and efficacy in reversing indicators of disease activity. Secondary outcomes involved changes in patient and parent-reported quality of life, assessed using PROMIS questionnaires.

**Results:**

Eliglustat was initiated at a mean age of 12.5 years (range: 6–17 years) and administered for a mean duration of 3.6 years (range: 1–7 years). All patients remained on treatment and exhibited sustained reductions in glucosylsphingosine (GlcSph) levels compared to baseline (*p* = 0.005). Other disease indicators demonstrated corresponding improvements. Adverse effects were limited to transient gastroesophageal reflux in 3/14 patients (21%). Serial electrocardiograms (EKGs) were normal. Growth and developmental milestones were appropriate for age in all patients. Patients and their parents reported a global improvement in quality of life.

**Conclusions:**

Eliglustat demonstrated significant clinical benefits in pediatric GD1 patients, as evidenced by reductions in GlcSph levels and other disease indicators. The therapy showed a favorable safety profile comparable to that observed in adults. These findings suggest eliglustat is a promising therapeutic option for pediatric GD1 patients, providing an effective alternative to ERT.

## Introduction

In Gaucher disease (GD), a biallelic mutation in *GBA1* results in defective lysosomal acid β-glucosidase (EC 3.2.1.45) and widespread cellular accumulation of lipids, glucosylceramide (GlcCer), and glucosylsphingosine (GlcSph), most conspicuously in tissue macrophages and immune activation (1). The multisystemic nature of GD causes significant morbidity in children, with manifestations ranging from hematological abnormalities to organomegaly and skeletal disease (2).

Recombinant enzyme replacement therapy (ERT) has been the cornerstone of GD treatment for decades, particularly in pediatric patients. Delivered biweekly via infusion, macrophage-targeted ERT effectively reduces GlcCer accumulation, reversing many disease manifestations and significantly improving quality of life (3). However, ERT presents notable challenges: the burdensome biweekly infusions impact both children and their families, and adverse events, such as infusion reactions or the development of neutralizing antibodies, can limit its effectiveness (4).

A key aspect of GD pathophysiology is metabolic inflammation driven by lipid accumulation. The buildup of GlcCer and GlcSph in macrophages orchestrates immune activation and induces UDP-glucose ceramide glucosyltransferase (UGCG, EC 2.4.1.80), the enzyme responsible for GlcCer synthesis (5, 6). This paradoxical upregulation exacerbates the metabolic defect, creating a cycle of lipid accumulation and inflammation. Eliglustat, a highly selective UGCG inhibitor, was approved in 2014 as a first-line oral therapy for adults with type 1 Gaucher disease (GD1). Studies have shown eliglustat to be well-tolerated, with efficacy comparable to or even exceeding ERT in certain measures, offering a convenient alternative for eligible patients (7, 8).

Despite its promise, pediatric patients have been underserved in clinical trials of eliglustat, limiting access to this oral therapy for children with significant barriers to ERT. A clinical trial investigating the use of eliglustat in children is ongoing (ClinicalTrials.gov ID: NCT03485677). In this study, we present real-world evidence for using eliglustat in pediatric GD1 patients with substantial barriers to ERT, providing insights into its safety, efficacy, and impact on quality of life.

## Methods

### Study setting

Patients were treated at a tertiary referral center specializing in Gaucher disease. All patients were referred due to symptomatic GD1 but faced significant barriers to receiving ERT. These included the presence of pan-neutralizing antibodies, the development of recurrent avascular necrosis despite ERT, needle phobia, or psychosocial challenges such as severe post-traumatic stress disorder (PTSD) from repeated infusions.

### Study population

The study included 14 consecutive pediatric patients diagnosed with GD1. The diagnosis was confirmed by low leukocyte acid β-glucosidase activity and pathogenic *GBA1* genotypes. The cohort comprised:

• Four patients (28%) developed pan-reactive, neutralizing anti-recombinant enzyme antibodies, rendering them ineligible for further ERT. One of these patients also developed AVN but was not counted in that category to avoid confusion.
• Four patients (28%) with severe PTSD from ERT infusions.
• Three patients (21%) who refused ERT initiation due to needle phobia.
• One patient who developed recurrent avascular necrosis (AVN) while on ERT.
• One patient who suffered from incapacitating social isolation and another for whom regular ERT infusions were unacceptably burdensome for normal school schedules.

Eliglustat therapy was initiated for all patients, with dosing based on pharmacogenomic CYP2D6 metabolizer status and body weight.

### Baseline evaluations

Comprehensive baseline assessments included:

• **Clinical and Laboratory Parameters:** Hemoglobin concentration, platelet count, complete metabolic profile, lipid profile, vitamin D, vitamin B12 and iron panel.
• **Organ Volumes:** Spleen and liver volumes were measured by abdominal ultrasound and expressed in multiples of normal (MN; 0.2% and 2.5% of body weight in kilograms, respectively).
• **Bone Health:** Lumbar spine bone density *Z* score, bone pain history, and symptoms of bone crises.
• **Biomarkers**: Serum levels of glucosylsphingosine (GlcSph) and chitotriosidase.
• **Cardiac Monitoring:** Baseline electrocardiograms (EKG) to evaluate QTc interval.

Due to the study's real-world nature and the need to minimize patient burden, not all data points could be consistently collected. However, all patients had pre- and post-treatment data for complete blood count (CBC), GlcSph levels, and EKGs.

### Outcome measures

1. **Safety Assessment:**
  Safety was evaluated by regularly monitoring adverse effects, including EKG assessments and patient-reported symptoms.
2. **Efficacy Assessment:**
  • **Hematological Parameters:** Changes in hemoglobin concentration and platelet count.
  • **Visceral Parameters:** Changes in spleen and liver volumes by ultrasonography.
  • **Biomarkers:** Serial measurements of serum GlcSph and chitotriosidase.
  • **Bone Health:** Monitoring bone pain, bone crises, and lumbar spine bone density *Z* scores.
  • **Growth Parameters:** Assessment of growth trajectories.
3. **Quality of Life:**
  Quality of life for patients and their parents was assessed retrospectively using the Patient-Reported Outcomes Measurement Information System (PROMIS) questionnaire.

Patients were analyzed in two cohorts: treatment-naïve patients and those who switched from ERT.

### Follow-up

Follow-up evaluations were conducted at 6–12 months intervals, with data collection spanning up to seven years.

### Statistical analysis

Statistical analyses were performed using R Studio and IBM SPSS Statistics. A paired *t*-test compared baseline and post-treatment values for hemoglobin concentration, platelet count, spleen volume, liver volume, GlcSph levels, and chitotriosidase levels. Missing chitotriosidase values were addressed using multiple imputations, and pooled analyses were performed.

We additionally performed a supplementary Linear Mixed Model (LMM) to account for repeated measures and variable follow-up durations among patients, using GlcSph levels as the dependent variable and time as the sole fixed effect. The LMM findings indicated a statistically significant decline in GlcSph over time (*p* < 0.001), consistent with the results from our primary analysis using paired parametric tests.

### Ethical considerations

Informed consent/assent was obtained from all participants or guardians to include data from their standard-of-care management in a longitudinal registry. The study adhered to the ethical principles of the Declaration of Helsinki.

### Patient demographics

Of the 15 pediatric patients who initiated eliglustat substrate reduction therapy (SRT), 14 patients diagnosed with Gaucher disease type 1 (GD1) remained on treatment and were included in the analysis. One patient with type 3 Gaucher disease (GD3) was treated with ERT/SRT combination therapy for massive calcific intrabdominal lymphadenopathy and intestinal malabsorption; SRT was withdrawn, and this patient was excluded from the analysis.

The *GBA1* gene mutations of the 14 included patients were distributed as follows:

• Seven patients (50%) were homozygous for p.Asn409Ser.
• Seven patients (50%) were compound heterozygous for p.Asn409Ser with:
  • p.Leu483Leu (*n* = 2)
  • 84GG (*n* = 2)
  • 217delC (*n* = 2)
  • IVS2 + 1 (*n* = 1).

Three patients (21%) were treatment-naïve, while the remaining 11 patients (79%) switched from ERT. The mean duration of eliglustat treatment was 3.6 years (range: 1–7 years) (Table 1).

**Table 1:** Demographic characteristics and *GBA1* mutations distribution.

	Mean *N*
Age at treatment initiation, mean (range)	12.5 years (6–17 years)
Sex, no. (%)	
Male	*n* = 6 (43)
Female	*n* = 8 (57)
Genotype, no. (%)	
p.Asn409Ser/p.Asn409Ser	*n* = 7 (50)
p.Asn409Ser/84GG	*n* = 2 (14)
p.Asn409Ser/IVS2+1	*n* = 1 (8)
p.Asn409Ser/p.Leu483Pro	*n* = 2 (14)
p.Asn409Ser/217delC	*n* = 2 (14)
Previous treatment history, no. (%)	
Switched from ERT to SRT	*n* = 11 (79)
Treatment naïve	*n* = 3 (21)
Duration of treatment, mean (range)	3.6 years (1–7 years)

ERT, enzyme replacement therapy; SRT, substrate reduction therapy.

### Demonstrative case: a patient with recurrent avascular necrosis (AVN) despite ERT

The patient was born in 2008 and presented at 2 years of age with chronic nosebleeds, easy bruising, and splenomegaly. Initially, diagnosis was of chronic liver disease prompting a liver biopsy which revealed Gaucher cells. Genetic testing at age 6 confirmed GD1 with biallelic *GBA1* mutations p.Asn409Ser and c.217delC. Initial evaluations revealed hepatosplenomegaly with liver and spleen volumes of 1.68 and 10.68 MN, respectively.

The patient was started on ERT at a dose of 60 units/kg every two weeks 6 years of age. However, the patient developed infusion-associated reactions, becoming refractory to premedication. Between ages 6 and 10, the patient experienced severe femur pain, eventually diagnosed as AVN via MRI, first in the left femur and later in the right femur. The patient had developed pan-reactive neutralizing antibodies after initiating velaglucerase and therefore had no response to switching taliglucerase or imiglucerase.

At evaluation in our center, the patient was wheelchair-bound, experiencing debilitating pain, with limited weight-bearing capacity. The patient was transitioned to eliglustat therapy to mitigate the progression of AVN.

**Figure 1:**
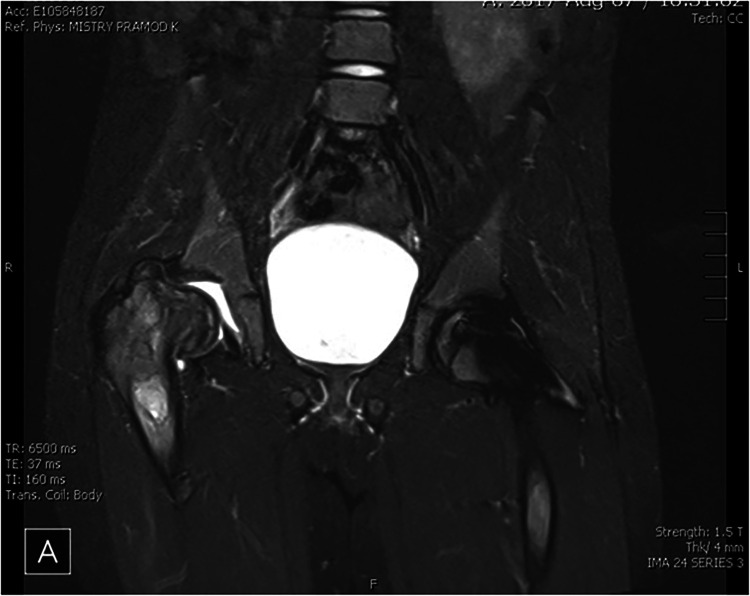
MRI pelvis of a patient with recurrent AVN on ERT (illustrative case). MRI of the pelvis showed chronic changes of avascular necrosis and flattening of the right femoral head with associated femoral neck widening. There is a loss of femoral head containment and visible right hip joint effusion. A geographic region of hyperintense STIR signal exists in the right proximal femoral diaphysis. There are postoperative changes of percutaneous pinning of the left hip limiting evaluation of the left femoral head and neck.

**Figure 2:**
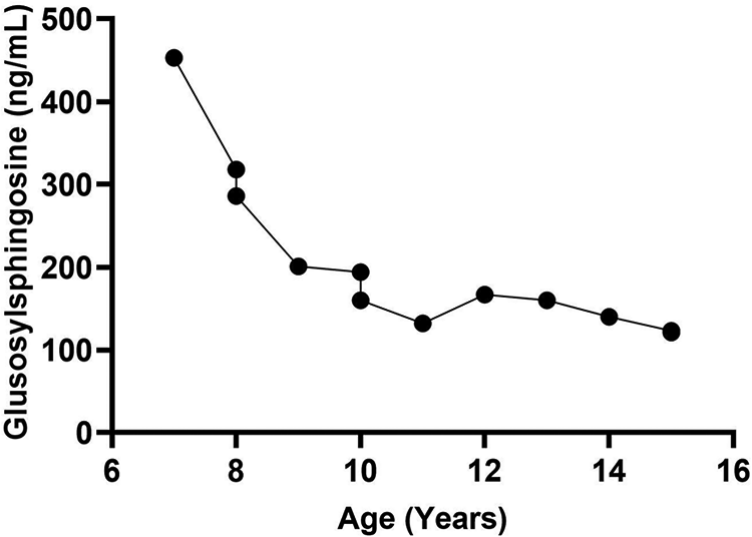
Glcsph trend in a patient with recurrent AVN on ERT. There were no new episodes of avascular necrosis on eliglustat SRT. The figure illustrates the reduction in disease biomarker of a patient with recurrent episodes of avascular necrosis on ERT. ERT was initiated at age 6. The patient was switched to treatment with SRT at age 8 years due to bone pain and development of neutralizing antibodies to enzyme. GlcSph, glucosylsphingosine; AVN, avascular necrosis; ERT, enzyme replacement therapy; SRT, substrate reduction therapy.

Over seven years of eliglustat treatment:

• No new bone crises or areas of osteonecrosis were reported (MRI showed no new lesions).
• Hematological and visceral parameters stabilized within the normal range.
• Serum glucosylsphingosine (GlcSph) levels drecreased consistently from 453 ng/ml to 121 ng/ml.

(Figures 1, 2 illustrate MRI findings and biomarker trends, respectively.)

### Biomarker response

Serum GlcSph levels, a reliable biomarker for monitoring GD activity (9), demonstrated a significant reduction:

• In treatment-naïve patients: 93% reduction (mean GlcSph decreased from 170.3 to 11.6 ng/ml; normal <1.0).
• In ERT-switch patients: 61% reduction (mean GlcSph decreased from 110.2 to 43.2 ng/ml).
• Across the entire cohort, the overall decrease was approximately 70% (*p* = 0.005).

**Figure 3:**
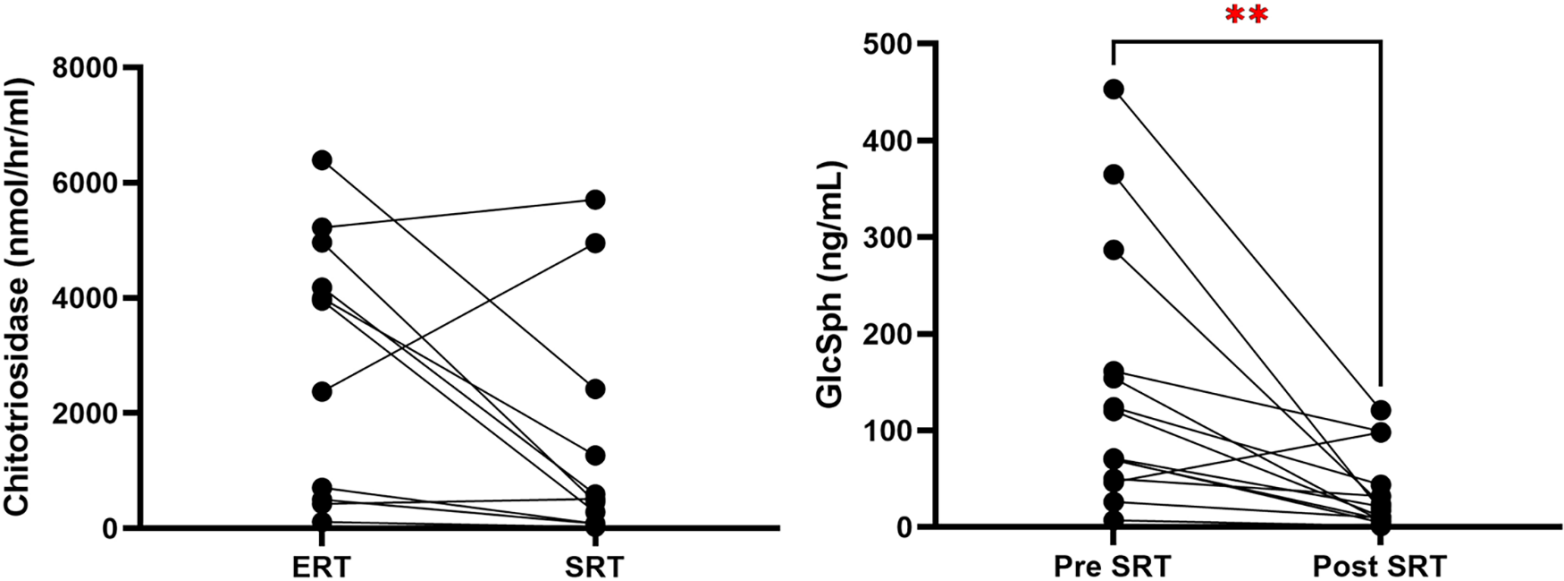
Reduction in chitotriosidase and GlcSph to eliglustat treatment in ERT to SRT switch and treatment-naive patients. This figure compares the reduction of chitotriosidase and GlcSph levels in pediatric Gaucher disease patients who were switched from ERT to SRT and in treatment-naive patients started on SRT. (*p* = 0.005). GlcSph, glucosylsphingosine; ERT, enzyme replacement therapy; SRT, substrate reduction therapy; GD3, type 3 Gaucher disease.

Chitotriosidase levels, normalized for *CHIT1* genotype, showed a 50% reduction from baseline (mean: 2,983 nmol/h/ml to 1,489 nmol/h/ml, *p* = 0.05) (Figure 3).

### Unusual treatment response

One patient (Patient 6) exhibited an apparent increase in GlcSph levels during follow-up, which may superficially appear as a failure of therapy. However, further investigation revealed that this was attributable to suboptimal treatment compliance, rather than a lack of efficacy of SRT. This underscores the importance of consistent treatment adherence in achieving optimal biochemical control in Gaucher Disease.

Another patient (Patient 14) with a concurrent diagnosis of metachromatic leukodystrophy (MLD) showed a dramatic decline in glucosylsphingosine levels, indicating excellent biochemical control of Gaucher Disease. However, despite this optimal response, the patient's hematological parameters were adversely affected. This can be attributed to recurrent infections and a history of sepsis, complications likely associated with the underlying MLD diagnosis.

These cases illustrate the importance of individualized patient evaluation to accurately interpret treatment outcomes, taking into account factors such as treatment adherence and concurrent diagnoses that may influence clinical and biochemical parameters.

### Hematological and visceral outcomes

Hematological and visceral parameters remained stable within therapeutic thresholds for GD1. Data for hemoglobin concentration, platelet count, liver, and spleen volumes are summarized in Table 2.

• Paired data for spleen volumes (*n* = 6) showed a mean reduction of 21.3%, from 3.75 multiples of normal (MN) (range: 1.16–7.94 MN) to 2.95 MN (range: 1.36–4.06 MN) following eliglustat therapy.
• Missing data on organ volumes resulted from logistical constraints (e.g., limited ultrasound access) and efforts to reduce the burden of MRI testing.

**Table 2:** Hematological and visceral values at baseline and post-treatment with eliglustat in treatment naïve and switched patients.

Patient	Age at start of treatment (years)	Treatment status	Gender/CYP2D6 Status	Total time on treatment (years)		Genotype
				ERT	SRT	
1	11	Treatment naive	M/EM	NA	6	p.Asn409Ser/p.Asn409Ser
2	14		F/IM	NA	2	p.Asn409Ser/p.Asn409Ser
3	11		F/IM	NA	3	p.Asn409Ser/p.Asn409Ser
4	8	Switched from ERT	F/NA	2	7	p.Asn409Ser/217delC
5	6		M/NA	2	7	p.Asn409Ser/217delC
6	15		M/EM	10	2	p.Asn409Ser/p.Leu483Pro
7	17		F/EM	9	2	p.Asn409Ser/p.Leu483Pro
8	14		M/EM	1	3	p.Asn409Ser/p.Asn409Ser
9	8		F/IM	1	2	p.Asn409Ser/84GG
10	17		M/IM	3	6	p.Asn409Ser/p.Asn409Ser
11	16		F/EM	8	5	p.Asn409Ser/IVS2+1
12	15		F/EM	5	5	p.Asn409Ser/p.Asn409Ser
13	16		F/NA	3	7	p.Asn409Ser/p.Asn409Ser
14	7		M/EM	1	2	p.Asn409Ser/84GG

Patient	Hemoglobin		Platelet count		Spleen volume		Liver volume	
	(g/dl)		(×10^9^/L)		(MN)		(MN)	
	Baseline	Current	Baseline	Current	Baseline	Current	Baseline	Current
1	12.4	15.3	248	272	2.39	3.01	0.97	1.15
2	12.7	13	168	161	5.1	NA	NA	NA
3	13.3	13.4	207	256	7.96	NA	1.32	NA
4	12.7	12.3	153	144	7.94	3.7	1.41	0.94
5	13.3	12.9	271	187	4.04	3.94	1.13	1.06
6	15.5	15.9	231	122	3.6	NA	NA	NA
7	14.1	12.9	215	180	3.2	NA	1.14	NA
8	12.5	14	125	201	2.15	NA	1.47	NA
9	13.4	13.2	270	313	1.16	1.63	NA	1
10	14.8	15.2	195	210	4.6	4.06	1.1	1.05
11	13.9	13.2	222	273	NA	2.7	NA	1.04
12	13.3	13.7	184	177	NA	NA	NA	NA
13	10.8	13.3	268	241	NA	NA	NA	NA
14	14.4	8.4	232	52	2.4	1.36	0.78	0.79

Patient	Chitotriosidase level		Lyso-GL 1 levels	
	(nmol/h/ml)		(ng/ml)	
	Baseline	Current	Baseline	Current
1	1,977.2	140	154	6.3
2	3,674.9	NA	286.9	24
3	499	84	70	4.5
4	6,391	2,419	453	121
5	5,217.4	5,704	50	32
6	2,372	4,954	46.41	98
7	54.9	13	26	10
8	4,964	461	365	17
9	3,995.6	1,268	161.3	99
10	705.8	84.3	69.3	8
11	426	514	71	21.1
12	729	NA	7.2	1.1
13	4,180	591	124	43.9
14	NA	NA	120	11

MN, multiples of normal organ size; EM, extensive metabolizer; IM, intermediate metabolizer; NA, not available.

### Bone outcomes

Over the mean follow-up period of 3.6 years, no episodes of new bone crises or osteonecrosis in the entire cohort.

• Bone density *Z*-scores (adjusted using the CHOP formula) were stable in three patients and improved in one patient.
• At baseline, four patients (33.3%) had low lumbar spine bone density, which remained stable or improved post-treatment.

### Quality of life

PROMIS questionnaires were retrospectively administered to 8 patients (57%) and their parents (57%). Improvements were noted across physical, mental, and social domains (Supplementary figures S1 and S2).

• No patient expressed a desire to return to ERT.
• Self-reported compliance with eliglustat exceeded 90%.

To mitigate bias, we administered PROMIS questionnaires to both patients and their caregivers and cross-referenced responses where discrepancies were noted.

### Adverse effects

Eliglustat was well-tolerated with no major adverse effects.

• The most common side effect was transient gastroesophageal reflux (3/14, 21%), managed with guidance on pill intake and not requiring treatment discontinuation.
• Serial EKG assessments remained normal, with no cardiac events (e.g., dizziness or syncope) reported.

## Discussion

The introduction of oral eliglustat substrate reduction therapy (SRT) for adults with type 1 Gaucher disease (GD1) in 2014 has transformed treatment paradigms (7). Dosed pharmacogenomically based on CYP2D6 metabolizer status, eliglustat has significantly improved treatment accessibility and adherence, offering an effective alternative to the lifelong biweekly enzyme replacement therapy (ERT) infusions (10). With the most extensive clinical trial program in Gaucher disease history, involving approximately 400 patients and over 1,400 cumulative patient-years of exposure, eliglustat has demonstrated excellent efficacy and tolerability in both treatment-naïve patients and those switching from ERT (11, 12). Compared to ERT, eliglustat reduces the treatment burden while directly addressing a key driver of pathophysiological mechanism of GD by inhibiting UDP-glucose ceramide glucosyltransferase (UGCG), thereby disrupting the cycle of lipid accumulation and inflammation (6).

Despite these advancements, eliglustat's use remains largely confined to adult populations due to delays in pediatric clinical trials (10–13). Pediatric patients with GD1 are often among the most severely affected, requiring timely access to effective therapies (14). Physiological differences, distinct pharmacokinetics, and ethical considerations in clinical trial design complicate the translation of adult data to children (15). Additionally, endpoints used in adult studies, such as MRI for organ volumetrics and marrow infiltration, are less practical in pediatric trials. Financial disincentives for pharmaceutical companies to prioritize pediatric studies further exacerbate these challenges. Consequently, the unmet therapeutic needs of children with GD1 remain substantial.

Barriers to ERT use among pediatric patients further highlight the urgency for alternative therapies. While infusion-related trauma and psychological morbidity, including PTSD, are widely recognized challenges, our study also observed a disproportionately high prevalence of neutralizing antibodies compared to the generally low prevalence reported in the broader population of patients treated with various ERTs (16). The presence of these antibodies can undermine ERT efficacy and complicate management, posing an additional hurdle for affected patients. It is important to clarify that the elevated prevalence of neutralizing antibodies in our cohort reflects the unique composition of our study population. As a tertiary referral center, we often attract patients with severe disease manifestations or atypical treatment responses, which naturally enriches our cohort with more complex cases compared to broader Gaucher populations.

Within this context, it is noteworthy that two of the four patients with neutralizing antibodies are siblings, suggesting familial concordance in immunological phenotypes. This observation aligns with established patterns in genetic diseases. When viewed in the broader context of our study population, comprising patients with severe disease and treatment resistance, the observed prevalence of neutralizing antibodies is consistent with expectations for a referral cohort.

Our study underscores the potential of eliglustat as an effective and well-tolerated oral therapy for pediatric GD1 patients. Significant reductions in serum GlcSph levels and other disease indicators, as well as lack of any new episodes of avascular necrosis, demonstrate eliglustat's clinical efficacy in our cohort. These findings align with existing data from adult studies and emphasize the broader benefits of eliglustat, including improved quality of life and treatment adherence. Patients and parents reported marked improvements across physical, mental, and social health domains, reflecting the reduced treatment burden and psychological relief from switching to oral therapy.

Eliglustat's favorable safety profile in pediatric patients, as observed in this series, mirrors outcomes from adult studies, reinforcing its role as a well-tolerated treatment option across age groups. No severe adverse events were noted, and common side effects, such as transient gastroesophageal reflux, were manageable. As assessed through serial EKGs, the absence of significant cardiac concerns further supports eliglustat's safety in pediatric use. These findings suggest that eliglustat could become a preferred therapeutic choice, particularly for pediatric patients facing challenges with ERT.

A multinational clinical trial of eliglustat in pediatric GD1 patients (ClinicalTrials.gov ID: NCT03485677) is currently underway to assess its safety, pharmacokinetics, and efficacy (17). In addition, emerging brain-penetrant SRT agents, such as venglustat, expand the therapeutic landscape for pediatric patients with GD3, reflecting a growing focus on personalized medicine in lysosomal disorders (18).

This case series highlights real-world experience with eliglustat in pediatric GD1 patients, providing important insights into its potential to improve clinical outcomes and optimize disease management.

Access to oral therapies like eliglustat represents a significant advancement in the treatment of pediatric Gaucher disease, offering an effective alternative to biweekly infusions and addressing critical unmet needs in this vulnerable population. Future studies, including long-term follow-ups and international registry analyses, will continue to elucidate the role of eliglustat in achieving personalized, patient-centered care for pediatric GD1 patients.

## Limitations

This study is not without limitations. Missing data for certain clinical parameters, such as spleen and liver volumes, posed challenges for comprehensive analysis. These data were not consistently collected to minimize the burden of testing in children, reflecting the real-world constraints of pediatric care. While this approach prioritized patient welfare, it limited the inclusion of key parameters in our statistical models. We acknowledge the limitations of retrospectively administered quality-of-life assessments. This approach was chosen due to the real-world nature of the study and the practical challenges of prospective QoL data collection in this patient cohort. While the findings provide valuable insights into perceived QoL improvements, we agree that prospective data collection in future studies would strengthen the validity of these results. Additionally, the study was conducted on a small population of 14 patients, which, while providing meaningful individual insights, may not fully represent the broader population of GD patients. Replication of these findings in larger, more diverse study cohorts or registries like ICGG will be essential to confirm the observed trends and enhance the generalizability of our results.

## Data Availability

The raw data supporting the conclusions of this article will be made available by the authors, without undue reservation.
