# Supplementary material for: Eliglustat substrate reduction therapy in children with Gaucher disease type 1
Source: Front Pediatr. 2025 Feb 27;13:1543136. doi: 10.3389/fped.2025.1543136 (PMC11903696; doi:10.3389/fped.2025.1543136)
Supplement: Supplementary file 1 [file Datasheet1.docx]

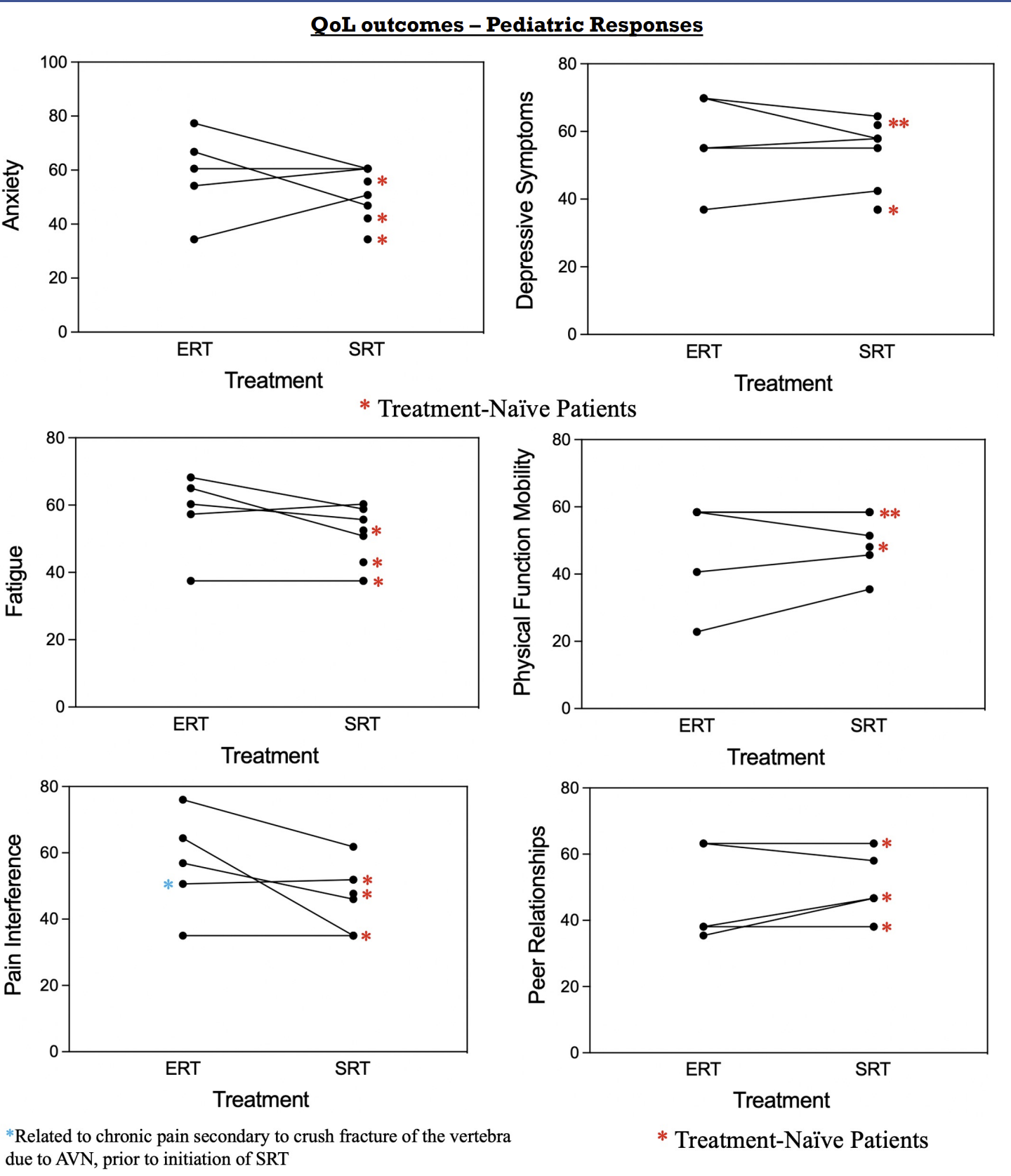


**Figure 1.1 Quality of Life Outcomes- Patient Responses**

**
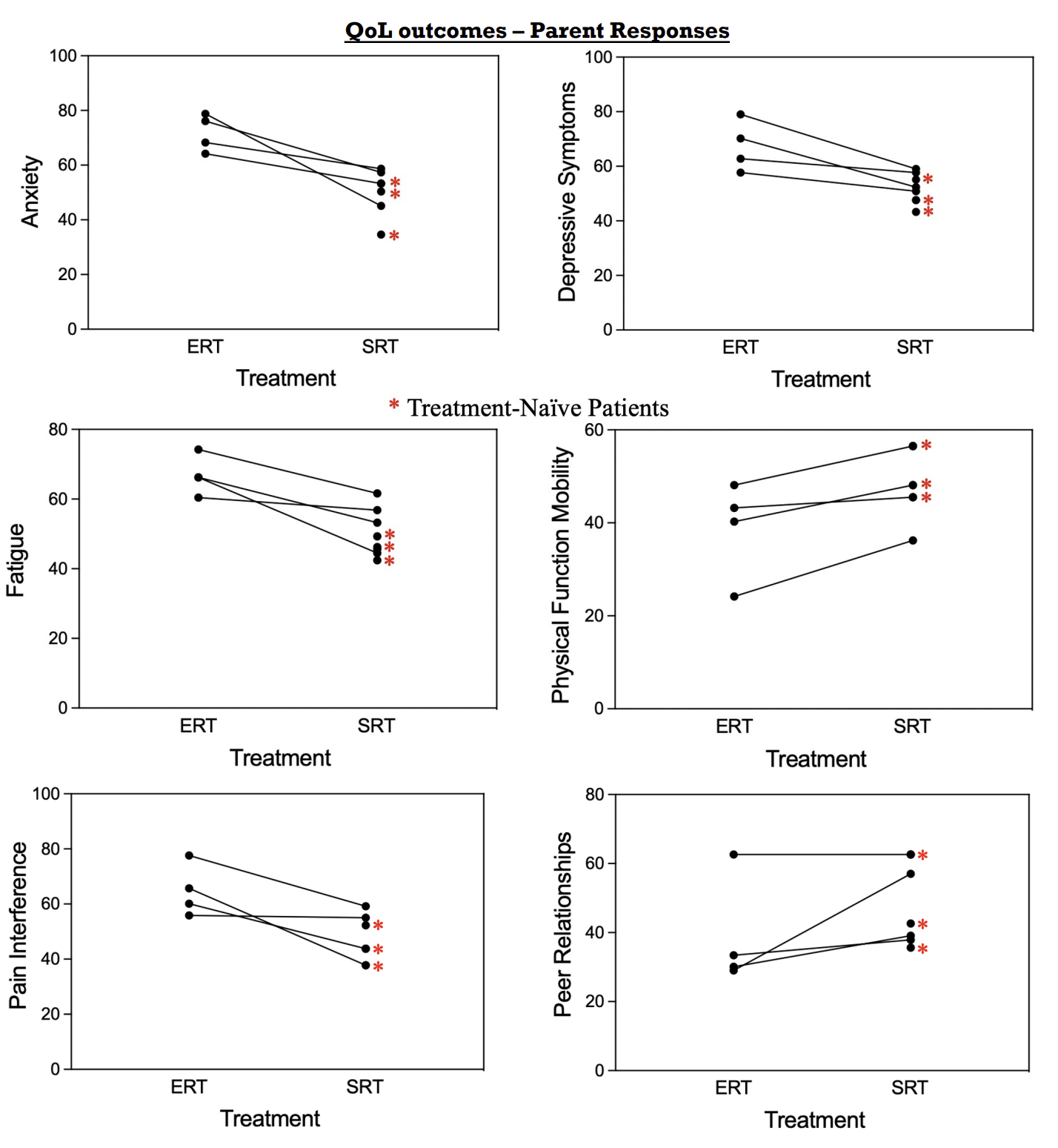
**

**Figure 1.2 Quality of Life Outcomes- Parent Responses**

**Table 1.1. PROMIS questionnaire data: patients**

| **Parameter** | **Anxiety** | | **Depressive Symptoms** | | **Fatigue** | | **Mobility** | | **Pain Interference** | | **Peer Relationships** | |
| --- | --- | --- | --- | --- | --- | --- | --- | --- | --- | --- | --- | --- |
| **Patient** | **ERT** | **SRT** | **ERT** | **SRT** | **ERT** | **SRT** | **ERT** | **SRT** | **ERT** | **SRT** | **ERT** | **SRT** |
| 1 | NA | 42.1 | NA | 36.9 | NA | 37.5 | NA | 58.4 | NA | 35 | NA | 38.1 |
| 2 | 60.5 | 60.5 | 55.1 | 57.9 | 60.3 | 55.7 | 58.4 | 58.4 | 56.9 | 46 | 38.1 | 38.1 |
| 3 | 77.4 | 60.5 | 69.8 | 57.9 | 68.2 | 58.8 | 22.8 | 35.5 | 76 | 61.8 | 38.1 | 46.7 |
| 4 | 34.4 | 50.8 | 36.9 | 42.4 | 37.5 | 37.5 | 58.4 | 58.4 | 35 | 35 | 63.2 | 63.2 |
| 5 | NA | 55.8 | NA | 61.9 | NA | 52.5 | NA | 58.4 | NA | 51.9 | NA | 46.7 |
| 6 | NA | 34.4 | NA | 36.9 | NA | 43 | NA | 48.1 | NA | 47.7 | NA | 63.2 |
| 7 | 54.2 | 60.5 | 55.1 | 55.1 | 57.3 | 60.3 | 58.4 | 51.4 | 50.6 | 51.9 | 63.2 | 58 |
| 8 | 66.8 | 46.9 | 69.8 | 64.5 | 65 | 50.8 | 40.6 | 45.7 | 64.4 | 35 | 35.4 | 46.7 |

**Table 1.2 PROMIS questionnaire data: parents**

| **Parameter** | **Anxiety** | | **Depressive Symptoms** | | **Fatigue** | | **Mobility** | | **Pain Interference** | | **Peer Relationships** | |
| --- | --- | --- | --- | --- | --- | --- | --- | --- | --- | --- | --- | --- |
| **Patient** | **ERT** | **SRT** | **ERT** | **SRT** | **ERT** | **SRT** | **ERT** | **SRT** | **ERT** | **SRT** | **ERT** | **SRT** |
| 1 | NA | 53.3 | NA | 47.6 | NA | 42.4 | NA | 45.5 | NA | 37.8 | NA | 35.6 |
| 2 | 68.3 | 58.7 | 62.8 | 57.7 | 66.2 | 53.2 | 43.2 | 45.5 | 60.1 | 43.8 | 33.4 | 37.9 |
| 3 | 76.1 | 57.4 | 70.2 | 52.4 | 74.2 | 61.6 | 24.2 | 36.2 | 77.6 | 59.2 | 29 | 57 |
| 4 | NA | 34.6 | NA | 43.3 | NA | 45.6 | NA | 56.5 | NA | 43.8 | NA | 62.6 |
| 5 | NA | 53.3 | NA | 55.1 | NA | 46.2 | NA | 48.1 | NA | 43.8 | NA | 42.6 |
| 6 | NA | 50.4 | NA | 47.6 | NA | 49.3 | NA | 56.5 | NA | 52.3 | NA | 62.6 |
| 7 | 64.2 | 53.3 | 57.7 | 50.9 | 60.4 | 56.8 | 48.1 | 56.5 | 55.9 | 55 | 62.6 | 62.6 |
| 8 | 78.8 | 45.1 | 79 | 59 | 66.2 | 44.5 | 40.3 | 48.1 | 65.7 | 37.8 | 30.1 | 39.1 |
